# Identification of Sex and Female’s Reproductive Stage in Commercial Fish Species through the Quantification of Ribosomal Transcripts in Gonads

**DOI:** 10.1371/journal.pone.0149711

**Published:** 2016-02-26

**Authors:** Iratxe Rojo-Bartolomé, Oihane Diaz de Cerio, Guzman Diez, Ibon Cancio

**Affiliations:** 1 CBET Research Group, Dept. of Zoology and Animal Cell Biology, Research Centre for Experimental Marine Biology and Biotechnology of Plentzia (PIE-UPV/EHU), University of the Basque Country (UPV/EHU), Areatza, z/g E-48620, Plentzia, Basque Country; 2 AZTI, Marine Research Division, Txatxarramendi, s/n E-48395, Sukarrieta, Bizkaia, Basque Country; The Ohio State University, UNITED STATES

## Abstract

The estimation of maturity and sex of fish stocks in European waters is a requirement of the EU Data Collection Framework as part of the policy to improve fisheries management. On the other hand, research on fish biology is increasingly focused in molecular approaches, researchers needing correct identification of fish sex and reproductive stage without necessarily having in house the histological know-how necessary for the task. Taking advantage of the differential gene transcription occurring during fish sex differentiation and gametogenesis, the utility of 5S ribosomal RNA (5S rRNA) and General transcription factor IIIA (*gtf3a*) in the molecular identification of sex and gametogenic stage was tested in different economically-relevant fish species from the Bay of Biscay. Gonads of 9 fish species (, Atlantic, Atlantic-chub and horse mackerel, blue whiting, bogue, European anchovy, hake and pilchard and megrim), collected from local commercial fishing vessels were histologically sexed and 5S and 18S rRNA concentrations were quantified by capillary electrophoresis to calculate a 5S/18S rRNA index. Degenerate primers permitted cloning and sequencing of *gtf3a* fragments in 7 of the studied species. 5S rRNA and *gtf3a* transcript levels, together with 5S/18S rRNA index, distinguished clearly ovaries from testis in all of the studied species. The values were always higher in females than in males. 5S/18S rRNA index values in females were always highest when fish were captured in early phases of ovary development whilst, in later vitellogenic stages, the values decreased significantly. In megrim and European anchovy, where gonads in different oogenesis stages were obtained, the 5S/18S rRNA index identified clearly gametogenic stage. This approach, to the sexing and the quantitative non-subjective identification of the maturity stage of female fish, could have multiple applications in the study of fish stock dynamics, fish reproduction and fecundity and fish biology in general.

## Introduction

There are more than 30000 fish species inhabiting a wide range of aquatic habitats worldwide [1], some of them being very important in the global economy and human diet. Moreover, as key integral members of ecosystems, fish are also becoming increasingly important sentinels of environmental health [2]. Some fish are also relevant laboratory models for the analysis of different developmental processes in vertebrates.

In recent years, as part of the policy to improve fisheries management, the estimation of maturity and sex ratio of fish stocks in European waters has become a fundamental requirement of the European Data Collection Framework (http://ec.europa.eu/fisheries/cfp/fishing_rules/data_collection/index_en.htm). Due to the overexploitation of fish from ocean sources some extractive fisheries have reached a plateau or are depleted. The impossibility of fish populations to breed on time is resulting in a significant loss of potential yield. In this scenario, it is imperative to study the reproduction biology of fish stocks, understand the population dynamics and the different changes that can occur in their life history [3]. Moreover, as reproduction largely determines productivity and the resilience of populations, it is crucial to estimate the quality and quantity of gametes. These are indicators of the reproductive capacity of commercial fish populations towards scientifically-based fisheries management [3, 4].

Presently, the allocation of maturity and sex in some fish species is laborious and requires a large number of sampled target organisms for visual and/or histological analysis of their gonads [3, 4]. This task is difficult when oceanographic campaigns do not coincide with the spawning season of the species under investigation and their gonads are poorly developed. In addition, many reproductive traits are highly variable and life history parameters (such as maturity at size or age, sex ratio, fecundity and spawning time/fraction) vary between species and populations or temporally within a single population [3, 4]. It should not be overlooked either that sex-determining genetic systems are highly diversified in teleosts: environmental factors play a major role in sex differentiation [1]. All of these factors together highlight the importance of studying the molecular and cellular mechanisms that drive sexual differentiation in fish. Most particularly, the growth and maturation of oocytes in females [5, 6] to determine the most efficient way to exploit and conserve different fish stocks.

On the other hand, fish biologists are increasingly applying molecular approaches in basic research studies to answer questions related to metabolism, reproduction, vertebrate development or to environmental stress and disease responses [1, 2, 7, 8]. Many molecular approaches need that the sex and the developmental stage of the particular individual under study are known, sometimes in the absence of the histological material necessary to obtain this knowledge. In these circumstances a molecular method towards fish sexing, carried out in the same biological material prepared for the specific methodological approaches of interest, would be advantageous [9].

Sex differentiation starts when primordial germ cells initiate their differentiation into either female or male germ line stem cells. During gametogenesis, oocytes and spermatozoids undergo diverse molecular and structural changes. Molecular changes during teleost oogenesis, for example, include variations in the level and nature of gene expression and in the accumulation of reserve substances (RNA, proteins, lipids, carbohydrates and hormones) necessary for early embryo development [7]. These molecules will be incorporated into the oocytes from surrounding ovarian follicular cells and from other organs such as the liver, which mobilises vitellogenin, a phospholipoprotein that is used in pollution monitoring as a biomarker of exposure to xenoestrogens in males and juveniles [8, 10].

5S ribosomal RNA (5S rRNA) is the smallest rRNA of the ribosome large subunit in eukaryotic ribosomes. Two types of tandemly-arranged 5S rRNA genes have been described for the anuran *Xenopus laevis*: one is expressed in somatic cells and testes; the other only in oocytes [11, 12]. Similar organization with two paralogue gene classes has been described in fish, such as the tench (*Tinca tinca*), hakes or mullets, amongst others [13–15]. Intench, 5S rRNA has been described as constituting, together with tRNAs, 90% of the RNA content of the ovaries [13, 16]. Whilst the precursor of the other ribosomal RNAs, 45S ribosomal RNA (45S rRNA) is transcribed by the RNA polymerase I (Pol I) in the nucleolus, 5S rRNA gene is transcribed by RNA polymerase III (Pol III) [12]. The activity of Pol III is regulated positively by the General transcription factor IIIA protein (Gtf3a), which also binds 5S rRNA transcript to assist in its stockpiling in the form of small 7S ribonucleoprotein particles in the cytosol [12, 17]. In the case of being fertilized, the accumulated 5S rRNA molecules are incorporated into the nucleolus to initiate ribosome assembly. Thus, interactions of 5S rRNA with Gtf3a and ribosomal proteins are crucial for the regulation of 5S rRNA biosynthesis [12]; this implies that, during expression of 5S rRNA genes in oocytes, a massive accumulation of *gtf3a* transcript occurs [2, 18]. It has been shown that, in early oogenesis, *gtf3a* constitutes more than 10% of the total cytoplasmic protein. This decreases 5–10 folds in later stages [12].

Based upon the different gene expression patterns in testis and ovary, these molecules can be diagnostic of the sex of the studied fish [2, 18]. We have shown previously that the accumulation of 5S rRNA and the high transcription levels of accompanying proteins in ovaries permit the identification of sex in thicklip grey mullets during their whole annual reproductive cycle [18]. Moreover, as 5S rRNA and *gtf3a* transcript levels in oocytes constitute also strong markers of xenoestrogenicity, identifying intersex testis in mullets inhabiting polluted estuaries [18]. It might be suggested that 5S rRNA and *gtf3a* constitute useful sex and oocyte maturity markers in teleost fish [2]. Very recently, Espigares et al. [9] used the accumulation of 5S rRNA to sex their experimental prepubertal European sea bass in the absence of biological material to carry out any histological analysis.

Within this context, the objectives of the present research are centered upon the study of 5S rRNA and *gtf3a* transcript levels as oocyte markers in several commercial teleost fish species captured in the Bay of Biscay (ICES Subareas VIII a, b, c and d; http://www.fao.org/fishery/area/Area27/en), then landed in the Basque Country fishing ports. Amongst the most important species in 2013 anchovy and pilchard constituted for the artisanal fleet 57% of the landings and 69% of the income or first sale value. European hake and blue whiting represented, for the bottom trawler fleet, 64% and 70% of the landings and income, respectively. Therefore, these species were chosen for their additional interest as economically relevant species in the Bay of Biscay, and due to the possible implications that studies on their reproduction could have for fisheries stock management.

## Materials and Methods

### Biological samples

All of the fish samples were obtained in the fish market of Ondarroa immediately after the fish were brought from the sea by commercial vessels that operate on working shifts of one day at sea. Samplings for each species depended on the season in which the different fisheries were open to the commercial fleet (Table 1). The species collected were: Atlantic mackerel (*Scomber scombrus*); Atlantic chub mackerel (*Scomber colias)*; blue whiting (*Micromesistius poutassou*); bogue (*Boops boops*); European anchovy (*Engraulis encrasicolus*); European hake (*Merluccius merluccius*); European pilchard (*Sardina pilchardus*); horse mackerel (*Trachurus trachurus*); and megrim (*Lepidorhombus whiffiagonis*).All individuals were dead on arrival at the harbour; they were kept on ice until dissection within 24 hours following their capture. On each sampling occasion around 20 individuals were measured and weighted, with the gonads being extracted (Table 1). Gonads were weighed and divided into two parts. One part was embedded in RNAlater® (Ambion, Life technologies), frozen in liquid nitrogen and then stored at -80°C until used further. The other part was fixed in 10% neutral buffered formalin containing 1% glutaraldehyde. All chemicals were of analytical grade and were obtained from Sigma-Aldrich (St. Louis, Missouri, USA) unless specified otherwise.

**Table 1:** Fish species studied in the present work with common and scientific names, capture locations and dates within the Bay of Biscay, number (n) of sampled individuals, sex ratio and histologically identified gametogenic stage according to the developmental stage of the oocytes in females (PV = previtellogenic, CA = cortical alveoli, AV = advanced vitellogenesis and Hy: hydrated).

Common name	Scientific name	Origin (ICES Subarea)	Capture month	(n)	Sex ratio (F/M)	Gametogenic stage
Atlantic chub mackerel	*Scomber colias*	VIII b	November 2011 & May 2014	14	8/6	PV
Atlantic mackerel	*Scomber scombrus*	VIII b	February 2012	13	7/6	AV
Blue whiting	*Micromesistius poutassou*	VIII c	May 2012	15	13/2	PV
Bogue	*Boops boops*	VIII b	May 2014	10	8/2	PV
European anchovy	*Engraulis encrasicolus*	VIII c	April & May 2012	31	15/16	AV & Hy
European hake	*Merluccius merluccius*	VIII b	March 2011	11	7/4	PV
European pilchard	*Sardina pilchardus*	VIII b	November 2011	12	7/5	Hy
Horse mackerel	*Trachurus trachurus*	VIII b	February 2012 & May 2014	24	7/17	PV
Megrim	*Lepidorhombus whiffiagonis*	VIII b	February, May & November 2012	31	26/6	PV, CA & AV

### Histological analysis

After 24 hours in the fixative gonads were dehydrated in a graded series of ethanol (70%, 90% and 96%) and embedded in methacrylate resin according to the manufacturer’s instructions (Technovit 7100; Heraeus Kulzer GmbH & Co. KG, Wehrheim, Germany). Resin sections (5 μm) were cut in a 2065 Supercut microtome (Leica Instruments GmbH, Nussloch, Germany) andstained with hematoxylin/eosin. Sex and gamete developmental stage were determined microscopically, following the gametogenic stage grading described by McDonough et al [19], and adapted for fish species with asynchronous developing gonads (Table 1).

### Extraction of total RNA, capillary electrophoresis and quantification of 5S/18S rRNA index

Total RNA was extracted from 50–100 mg of tissue using TRIzol® (Invitrogen, Carlsbad, California, USA) and following the manufacturer´s instructions. Obtained RNA was purified using Qiagen RNeasy kit (Qiagen, California, USA) after a DNase digestion step (RNase-Free DNase Set, Qiagen). After purification, the same amount of RNA (250–500 ng), as estimated through absorbance at 260 nm, (good quality RNA established at 260/280 and 260/230 ratios around 2) was loaded in an Agilent RNA 6000 Nano Kit Bioanalyzer (Agilent Technologies, Santa Clara, California, USA). Electropherograms provided by the Bioanalyzer were used to quantify the concentration of the bands corresponding to 5S rRNA and 18S rRNA in each sample. The Time Corrected Area of each peak was used to calculate the 5S/18S rRNA ratio. When the presence of one of the rRNAs was below the levels of detection of the machine, 5S rRNA in the case of a few males and 18S rRNA in the case of a few females, a 0,1 value was given to each sample instead of 0 (the lowest recordable value was 0,2). The logarithm of the ratio was calculated in order to develop an index that allowed clear visualization of the differences between testes and ovaries.

This study was also extended to the gonads of 5 female and 5 male adult zebrafish (*Danio rerio*; UB Tubingen) from our own stock. Zebrafish individuals were euthanized by an overdose of MS-222 (tricaine methane-sulfonate) following the protocol authorised by the ethics commission of the University of the Basque Country CEEA/337-2/2014/ORTIZ ZARRAGOITIA. Gonads were dissected, immersed in RNA Later® (Ambion) and immediately frozen in liquid nitrogen, and stored at -80°C until RNA extraction. These zebrafish samples served as controls, whose RNA profiles could be compared with those extracted from the commercial fish species that were not dissected directly upon capture.

### *gtf3a* cloning and sequencing

2 μg of total RNA from each individual gonad were used for cDNA synthesis with AffinityScript Multiple Temperature cDNA Synthesis Kit (Agilent Technologies) using random primers.

*gtf3a* mRNA fragments were amplified using conventional PCR and employing 0,8 mM degenerate primers: forward 5´-TGGARGCTCATCTKTGCAAACACAC-3´ and reverse 5´-GTCYCCDCAGGCTYTCCTTCATG-3´. Primers were designed through alignment (Clustalw2, http://www.ebi.ac.uk/Tools/msa/clustalw2/) of piscine *gtf3a* sequences available in Ensembl and GenBank and searching for highly conserved nucleotide regions. Properties of designed primers were checked employing the IDT OligoAnalyzer Tool (https://eu.idtdna.com/calc/analyzer).

The amplification was run with commercial Taq DNA Polymerase, recombinant Kit and 100 mM dNTP Mix (Invitrogen) for 35 cyclesin a 2720 Thermal Cycler (Applied Biosystems, Carlsbad, California, USA). PCR procedure was as follows: 94°C for 2 minutes, denaturation at 94°C for 30 seconds, annealing at 58°C (Tm) for 30 seconds, elongation at 72°C for 8 minutes and finally 72°C for 8 seconds. PCR products were stored at 4°C until they were analysed by electrophoresis in ethidium bromide stained agarose gels (1,5%) and sent to the SGIker Sequencing Service of the University of Basque Country for sequencing. Once sequenced, fragments were aligned to obtain a consensus sequence and analyzed using CAP3 (http://pbil.univ-lyon1.fr/cap3.php) and ClustalW2 tools.

All sequences obtained have been published in GenBank for Atlantic chub mackerel (JQ928632); Atlantic mackerel (JQ928631), blue whiting (KC191719), European hake (JQ928630), European pilchard (JQ928634), horse mackerel (KC191721) and megrim (JQ928633), and.

### Real time PCR

cDNA obtained from megrim was quantified in the Synergy HT Multi-Made Microplate Reader (BioTek, Winoosky, USA) by Quant-it^TM^ OliGreen^®^ stain (Invitrogen). The quantification was performed in a reaction volume of 100 μl with a theoretical cDNA concentration range of 0,02–0,2 ng/μL, at 485/20 nm excitation and 528/20 nm emission wavelengths. Real PCR input cDNA concentration was calculated using the high-range standard curve according to the manufacturer’s instructions.

5S and 18S rRNA transcript levels were quantified in megrim females by SYBR Green^®^ qPCR (Roche, Basel, Switzerland). qPCR was conducted in triplicates using a 7300 Applied Biosystems Thermocycler (Applied Biosystems). The 20 μl PCR reaction mixture consisted of 10 μl of 2× SYBR® Green PCR master mix, appropriate concentration of 5S and 18S rRNA primers diluted in RNase-free water (final primer concentration: 12,5 nM) and a 2 μl cDNA template. 5S rRNA primers are protected under Spanish patent P201130778 and international patent PCT/ES2012/070343. 18S rRNA primers were: forward 5’-CCTTTTAACGAGGATCCA-3’; and reverse 5’-ACGGCTACCACATCCAA-3’ (Tm: 55°C). qPCR procedure was as follows: 50°C for 2 minutes, 95°C 10 minutes, then 40 cycles of denaturation at 95°C for 15 seconds and annealing temperature for one minute. Dissociation stage was added at the end with 95°C for 15 seconds, one minute in annealing temperature, 95°C for 15 seconds and a final step of 60°C for 15 seconds.

*gtf3a* transcript levels were quantified also in megrims using Probe #76 from the Universal Probe Library (Roche) according to the manufacturer’s instructions; for 40 cycles and with 500 nM of the specific primers designed in Universal ProbeLibrary Assay Design Center (Roche): forward 5’-CAGCACCAAGAGAAGCGATA-3’ and reverse 5’-TGGTTCCTCTTGTTGAAATCC-3’, Tm: 60°C. All gene transcription results were normalized with the amount of cDNA charged in the qPCR (input cDNA, [20]) using for that a ΔCT formula adapted from the ΔΔCT normalization method:

$$RQ={Log}_{2}\left[\frac{{\left(1+Eficiency\right)}^{\left|\Delta CT\right|}}{ng\;cDNA}\right]$$

Where Δ*CT* = *CT sample* − *CT plate internal control*

### Statistical Analysis

The statistical analyses were undertaken using SPSS (SPSS Inc., Chicago, Illinois). Significant differences in RNA levels between both sexes were evaluated by the non-parametric Mann-Whitney U-test. Significant differences in RNA levels, when ovarian developmental stages (and testis) in megrim were compared, were analysed applying one-way ANOVA. Significant differences between groups, in terms of qPCR gene transcription levels, were evaluated using the non-parametric Jonckhere-Terpstra test when *a priori* ordering was assumed for more than two independent samples (PV>CA>AV). In all cases, significant differences were established at *p*< 0.05. Minimal data set for generation of figures and statistical analysis is provided in S1 and S2 Tables.

## Results

### 5S rRNA in gonads: identification of sex in teleost fish

**Fig 1:**
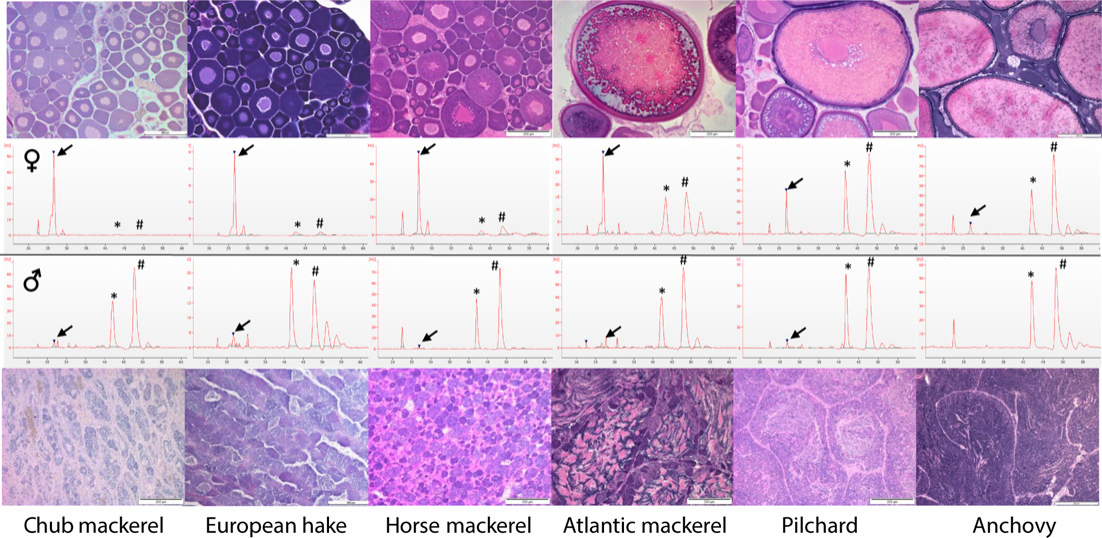
Representative electropherograms of gonad total RNA of different fish species. Electropherograms of gonad total RNA from previously histologically sexed male (bottom) and female (top) Atlantic chub mackerel, European hake, horse mackerel, Atlantic mackerel, European pilchard and European anchovy. Electropherograms and histological micrographs (in all cases scale bars = 200 μm) are representative of all the individuals sampled and analysed per species. Oocytes in ovaries of chub mackerel, hake, and horse mackerels were at previtellogenic stage, in advanced vitellogenesis in Atlantic mackerel and hydrated in the case of pilchard and anchovy. Notice that 5S rRNA in ovaries was relatively the most prominent in the species where ovary only displayed previtellogenic oocytes. Arrows indicate the 5S rRNA peak. * = 18S rRNA peak, # = 28S rRNA peak.

The electropherograms obtained from gonads of commercially-relevant fish species showed different RNA patterns when comparing total RNA extracted from adult testes and ovaries. No signs of RNA degradation, clearly observable in Agilent RNA chips run in the 2100 Bioanalyzer, were observed in any of the samples, In the case of ovaries, the relative 5S rRNA signal was always higher than in the testes. When ovaries presented oocytes in previtellogenic stages the 5S rRNA peak was higher than those of 18S and 28S rRNAs, which are prevailing in all eukaryotic cells (Fig 1, S1 and S2 Figs). The relative amount of 18S and 28S rRNA in ovaries becomes more similar to that of the testis the more advanced they are in the oogenesis process (Fig 1).

### 5S/18S rRNA index: identification of oogenesis stage

**Fig 2:**
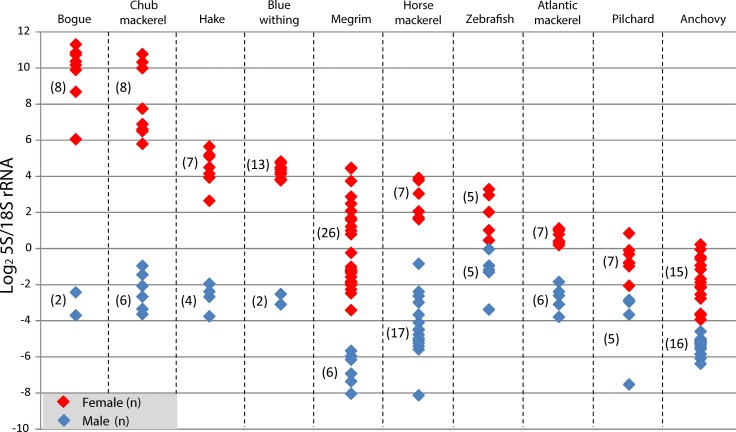
5S/18S rRNA index in the gonads of different commercial fish species. Red dots identify ovaries and blue dots identify testes. Each dot corresponds to an individual and numbers between parentheses identify the number of individuals analysed per sex. Species are ordered from left (previtellogenic oocytes) to right (hydrated oocytes) as follows: bogue, Atlantic chub mackerel, hake, blue withing, megrim, horse mackerel, zebrafish, Atlantic mackerel, pilchard and anchovy. Female samples for megrim and anchovy displayed ovaries in different developmental stages (see Fig 3 and S3 Fig). Index values between both sexes were statistically different in all the 10 species studied (Mann-Whitney, p< 0,05)

**Fig 3:**
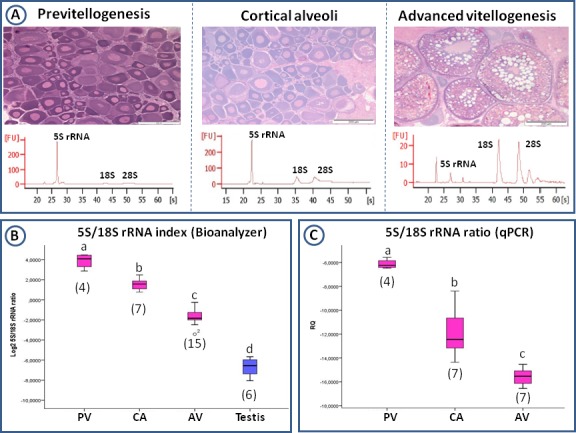
Total RNA electropherograms and 5S/18S rRNA index in the gonads of megrims. (A) Gonad histology (scale bars = 200 μm) and electropherograms representative of total RNA distribution in megrim ovaries (captured in February, May and November) in different stages of oogenesis (previtellogenesis -PV-, cortical alveoli -CA- and advanced vitellogenesis -AV-). [FU] = Fluorescence, [s] = Time in seconds. The peak at around 20 seconds corresponds with the Agilent marker. 5S rRNA band appears between 25 and 30 seconds, 18S rRNA band around 40 seconds and 28S rRNA in between 45 and 50 seconds. (B) 5S/18S rRNA index in ovaries in different developmental stages and in testis as quantified from electropherograms. (C) 5S and 18S rRNA index as obtained after qPCR analysis of the levels of transcription of both genes in ovaries in different developmental stages. Numbers between parentheses indicate the number individuals analysed per group. Different letters indicate significant differences among pairs of means (one-way ANOVA, p<0,05 in B, Jonckheere-Terpstra, p<0,05 in C).

The quantification of the concentrations of 5S and 18S rRNA in the electropherograms permitted the calculation of a 5S/18S rRNA index. This index separated males from females in all of the species being studied (Fig 2), including the model species zebrafish. Zebrafish from our own stock, allowed obtaining freshly prepared gonad samples. Electrophoretic profiles corroborated that profiles observed with RNA extracted from organs harvested from commercial fish species upon collection at the harbour were not the result of RNA degradation. When females were captured during advanced oocyte developmental stages (Atlantic mackerel, Euroepan anchovy and pilchard and zebrafish or) the ratio was significantly lower than in those captured during resting/previtellogenic stages (blue whiting, bogue, chub and horse mackerel, European hake). The index threshold value, which permited sex discrimination, was species and gametogenic stage dependent (Fig 2). This observation was confirmed in megrim where, as a result of three different samplings, females with oocytes in previtellogenic, in cortical alveoli and in advanced vitellogenic stages were available (Fig 3). The 5S/18S rRNA index grouped vitellogenic ovaries together and separated them from previtellogenic ones (Figs 2 and 3B). Megrims in advanced vitellogenesis showed index levels closest but different to males (Fig 3B), as a consequence of the appearance of 18S rRNA. Similarly, two different samplings were carried out with anchovies. In both cases, females were captured during the spawning period but, in one case, oocytes were completely mature and index values were closest to those of males. In the other case, oocytes had not yet begun final maturation, with a higher 5S/18S rRNA index (S3 Fig).

To corroborate that we were really measuring 5S and 18S rRNA levels in the electropherograms and no anything else, specific transcription was analyzed through qPCR in megrim. The 5S/18S rRNA index generated matched perfectly the results obtained with measurements on electropherograms, distinguishing ovaries on the basis of their gametogenic stage (Fig 3C).

### *gtf3a* in commercial fish species; cloning and sex-specific transcript levels

**Fig 4:**
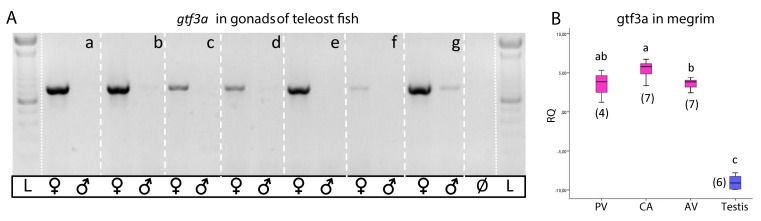
*gtf3a* transcription levels in ovaries and testis of fish. (A) Agarose gel electrophoresis of *gtf3a* fragments (around 700 nucleotides in length) amplified in one ovary and one testis through conventional PCR for 35 cycles. a: Atlantic mackerel; b: *chub mackerel*; c: megrim; d: blue whiting; e: hake; f: pilchard; g: horse mackerel. Developmental stages of ovaries as in Table 1, megrim in advanced vitellogenesis. Ø = no template control, L = Standard 100 bp (Invitrogen). (B) Box plots representing *gtf3a* transcript levels in megrim ovaries in different developmental stages (PV = previtellogenic, CA = cortical alveoli, AV = advanced vitellogenesis) and in testis. Numbers between parentheses indicate the number of individuals analysed per group. Different letters indicate significant differences (Jonckheere-Terpstra, p<0,05) between groups. Data normalized to cDNA ng per sample.

Use of degenerate primers permitted the amplification and sequencing of *gtf3a* in ovaries of 7 of the studied fish species using conventional PCR. cDNAs used to obtain these sequences were produced with RNA extracted from ovaries with previtellogenic (Atlantic chub and horse mackerel, blue whiting and hake,), advance vitellogenic (Atlantic mackerel and megrim) and hydrated (pilchard) oocytes. All of the fragments published are 523–707 nucleotides in length (S3 Table). *gtf3a* was transcribed strongly in the ovaries in all the species (developmental stages for each species as before and in Table 1), whilst nearly no transcription was detected in testes (Fig 4A). It was not possible to clone a *gtf3a* orthologue in anchovy and bogue.

qPCR analysis of *gtf3a* was performed on megrim gonads. The results obtained showed significant differences between females and males (Fig 4B). Besides, ovaries in previtellogenic and cortical stages presented the highest *gtf3a* transcript levels, coincident with the oogenesis stages in which 5S rRNA production was at its highest and 18S rRNA at its lowest (Fig 4B). A significant down regulation of g*tf*3a was recorded when ovaries reached advanced vitellogenesis.

## Discussion

The results of the present study revealed marked sex-related differences in 5S rRNA and *gtf3a* transcript levels in gonads of fish species commercially-relevant in the Bay of Biscay, irrespective of their reproductive stage. It has been shown that the electrophoretic analysis of total RNA extracted from gonads is sufficient to identify sex due to the high production and accumulation of 5S rRNA in the oocytes. This approach to sex fish only requires extraction of total RNA and running an electrophoresis so it is cheap, not as skill demanding as expert histological analysis and it requires a minimum amount of tissue. In 7 of these species *gtf3a* was sequenced and its quantification through PCR also permitted sex identification in all of the species analysed. Using this knowledge an index based on the quantification of 5S rRNA and 18S rRNA levels in electropherograms was developed; this allowing non-subjective female gametogenic stage identification in all the studied fish species.

### 5S rRNA: molecule useful in sex identification

Evidence that 5S rRNA gene is expressed always in higher amounts in ovaries than in testes in fish, independent of gametogenic stage during the seasonal reproductive cycle, was presented firstly in thicklip grey mullets [18]. This observation was hypothesised as corresponding to a mechanism to accumulate rRNA intermediates that should be available in the oocyte to, in the event of being fertilised, allow rapid assembly of ribosomes and sustain protein production during early embryo development [2, 18]. It was confirmed here that this observation can be generalized to other teleosts, at least temperate marine fish species plus zebrafish, as 5S rRNA gene was also highly expressed in the ovaries of all of the commercially-relevant fish species that were studied. Therefore, it was confirmed that high levels of 5S rRNA in gonads could be diagnostic of teleost fish sex, identifying females independent of the period within their reproductive cycle and of the species studied. Such a molecular approach could provide an easy tool for the allocation of sex in other fish species, which is otherwise somewhat laborious and not easy when samplings do not coincide with the spawning season [18], when enough gonad tissue is not available or when tissue has not been processed for histological analysis [9]. For instance, in molecular biology laboratories where transcriptomic studies are carried out, electrophoretic analysis of RNA quality should be standard procedure, giving this additional information on the sex of the fish [9]. It should be taken into account though, that this is a post-mortem approach and that requires gonad dissection. Moreover this sexing approach is only effective when sex differentiation has already occurred, as high 5S rRNA transcription is the consequence of having oocytes.

5S rRNA accumulation in fish ovaries has been shown already elsewhere by other authors in different teleost fish [13, 16]. Very recently it was also proved in prepubertal European sea bass [9]. This observation might have important implications in transcriptomic studies using fish ovaries. The RNA Integrity Number (RIN), which has become the gold standard method to evaluate total RNA quality [21], proves to be not useful to analyze RNA extracted from ovary due to the prevailing presence of 5S rRNA and other small-sized RNA molecules [22]. For example, in sea bream (*Diplodus puntazzo*), and due to the presence of prevailing RNA peaks around 100 nucleotides, Manousaki *et al*. [23] concluded that it is not possible to use total RNA electrophoresis and the RIN number to estimate the RNA quality in ovaries that are in early developmental stages. Similar conclusion was reached by Kroupova et al. [24].

The fish species studied in this work presented different reproductive strategies, which have strong implications in the analysis of their fecundity. Following the classification proposed by Murua *et al*. [4] females can be classified as asynchronous indeterminate batch spawners (Atlantic chub and horse mackerels, blue whiting and European anchovy, hake and pilchard,), asynchronous determinate batch spawners (Atlantic mackerel) and group-synchronous batch spawners (megrim). No data are presented here on any synchronous-determined total spawner, but the previous study by Diaz de Cerio *et al*. [18] focused on thicklip grey mullet, which presents this reproductive strategy. In all cases, 5S rRNA distinguished males independent of the reproductive strategy.

### *gtf3a*: sex specific transcription levels

*gtf3a* was cloned and sequenced in 7 species of the 9 studied; and its high transcription levels in ovaries in comparison to testes also allowed easy identification of sex. In this way, *gtf3a* could be considered also a potent molecular sex marker for teleost fish.

In all of the cells, ribosome biosynthesis monopolizes up to 80% of the cellular transcription activity and requires the synthesis of RNAs by three nuclear RNA polymerases: Pol I, which produces the precursor of 5.8S, 18S and 28S RNAs; RNA polymerase II(Pol II), which produces all mRNAs including those that encode ribosomal proteins; and Pol III, responsible for 5S rRNA gene transcription [25]. Activity of Pol III requires Gtf3a, which has the ability to bind both 5S rRNA gene and 5S rRNA itself. Firstly, it recognizes and binds the promoter sequence 5S rRNA gene helping in the assembly of the transcription machinery of Pol III [17]. Subsequently, Gtf3a binds 5S rRNA for stockpiling in the cytosol in the form of 7S ribonucleoprotein particles, formed by one molecule of 5S rRNA and one Gtf3a protein molecule [17].

While low *gtf3a* levels appear to be a common feature of somatic cells, extremely abundant *gtf3a* transcript levels have been long known in amphibian oocytes [11, 26]. Levels of *gtf3a* mRNA mirror those of 5S rRNA; in *Xenopus these* are about 1 million times higher in oocytes than in somatic cells [17]. Consistent with this observation, it has been reported that Gtf3a predominantly binds the oocyte-type and not the somatic-type 5S rRNA in *Xenopus*, even when both sequences only diverge in three nucleotides [11]. Moreover, *gtf3a* is overexpressed early in oogenesis, constituting even 10% of the total cytoplasmic protein in anurans, then decreasing 5-10-fold by later stages [17, 27].

Little is known about *gtf3a* transcription dynamics in fish [2]. Functional aspects of Gtf3a were studied only in catfish (*Ictalurus punctatus*) oocytes, showing a similar capacity to bind oocytic 5S rRNA [28]. Diaz de Cerio *et al*. [18] demonstrated, through qPCR analysis for the first time in fish, that *gtf3a* transcriptional regulation resembles that of 5S rRNA in ovaries. Interestingly, *gtf3a* appeared *also* in lists of zebrafish and flounder (*Paralichthys olivaceus*) ovary enriched transcripts [29, 30]. Our qPCR results clearly identified *gtf3a* early expression in megrim oocytes. *gtf3a* transcript levels were at their highest in the early stages of ovary maturation at cortical alveoli stage, decreasing during vitellogenesis, as in *Xenopus* [26]. No reference genes were used to normalize the target gene transcription data; instead, the amount of input cDNA per sample was used [9]. The traditional qPCR normalization method includes the use of reference or housekeeping genes with presumably invariant levels of transcription in each particular experimental system [31]. Ovaries suffer profound changes in terms of cell composition, physiological status, hydration level, or meiotic stage; as such their RNA content differs in amount and composition throughout growth and maturation. No valid reference gene exists in these circumstances. Libus and Štorchová [32] and Mittelholzer *et al*. [22] proposed the use of total amount of cDNA to normalize qPCR data in fish gonads. In addition, Filby and Tyler [20] used also ΔCT method with input cDNA amount for normalisation purposes when studying the usefulness of reference genes in fish ovaries. Also, De Santis and Smiths-Keune [33] used cDNA for the normalization of muscle data in barramundi (*Lates calcarifer*). These investigations concluded that this new normalization approach may produce the most biologically-valid results when studying very dynamic tissues where reference genes are not stably expressed. Similar conclusions were reached by other authors when studying different tissues and organs [34, 35].

### 5S/18S rRNA index: female developmental stage identification

5S rRNA quantification provides an easy way to sex teleost fish species in a comparative basis (comparing a female and a male individual on the same electrophoresis) [2, 9, 17]. However, it would be useful to set threshold expression levels to unequivocally identify sex. For this purpose, a 5S/18S rRNA index was developed after quantifying the concentration of 5S and 18S rRNAs in the electropherograms provided by the Bionalyzer RNA nanochips. 18S rRNA was selected for the quantification of the index since 28S rRNA could be more prone to cleavage because of its bigger size. The logarithmic transformation of this ratio allowed improved visualization of the differences between both sexes.

The index did not only allow absolute identification of sex without the need of comparing two individuals but also ranking the development stage of the oocytes present in the ovary due to changes in the relative amount of RNA species throughout oogenesis. Kroupova *et al*. [24] analyzed in detail the stage-dependent RNA composition in roach (*Rutilus rutilus*) ovaries, concluding also that during primary growth and early cortical alveoli stages smaller-size RNAs were accumulated. In megrim females the index was highest in previtellogenic females and lowest in advanced vitellogenic ones. Thus, a developed 5S/18S rRNA index could constitute a new approach to study the reproductive stage in females, independent of their reproductive strategy. It is true that as 5S rRNA predominated in previtellogenic oocytes this index should work better in identifying the reproductive stage in ovaries with synchronous development. In any case, in the present investigation, where most species studied displayed an asynchronous development with different type of oocytes mixed within the ovary, the index proved to be useful in grading the maturity of females. Thus, the developed index could be of considerable assistance in the characterization of reproductive potential; this is extremely important in the study of fish stocks dynamics [5] and fish biology research in general.

The data presented here could also contribute to studies of fish fecundity, essential to develop an understanding of the stock and recruitment relationship in fish, or in the assessment of spawning stock biomass. Nowadays, methodological approximations to fish population fecundity studies depend on the spawning strategy of females; likewise, then are very laborious and required skilled histological capabilities [5, 36, 37]. In any case, the methodology presented here could be a very useful approach for laboratories that use molecular approaches in their research, and that could need of proper identification of sex and reproductive stage of their samples as supporting parameter [9].

### Why did 5S rRNA and 18S rRNA relative amounts change during the development of the oocytes?

Previous studies on *Xenopus* have shown that during early previtellogenic stages rRNA genes are amplified to a final content of ~2x10^6^ per oocyte; this provides sufficient rRNA gene templates to produce 1x10^12^ ribosome particles per mature oocyte [38]. Despite this amount of rRNA genes, the transcription of 45S rRNA (precursor of 5.8S, 18S and 28S rRNA) takes place at very low levels in previtellogenic stages but increases dramatically in vitellogenic stages. 18S and 28S rRNA transcription occurs in response to the activation of Pol I during vitellogenesis [12]. On the contrary, Pol III activity is maximal in the previtellogenic stages in *Xenopus* [12]. The results described here, together with those presented for roach ovaries [24], would indicate the same trend as in the case of anurans. Ovaries at previtellogenic and cortical alveoli stages would accumulate small-sized RNAs. It could be hypothesised that, during early oogenesis, energetic investment in reproduction takes a decision towards the least energetically demanding production of 5S rRNA. When reproduction is envisaged to occur under favourable conditions, oocyte secondary growth is initiated. The real energetic investment in reproduction commences with the production of the large ribosomal RNA molecules and vitellogenesis. Thus, the 5S/18S rRNA index changed during differentiation, with the inactivation of Pol III activity (observed in megrim as decreased *gtf3a* transcript levels in advanced vitellogenic ovaries) and activation of Pol I at vitellogenesis.

Overall, the results presented here confirmed that 5S rRNA and *gtf3a* are useful molecular sex markers in teleost fish; at least in the ones studied to date. Moreover, these molecular markers could be used easily to establish the gonad developmental stage in females. Presently attempts are being made to develop a fish sexing molecular kit, with technological transfer potential, based upon the qPCR assessment of the transcriptl levels of 5S rRNA. This molecular approach could be relevant for the study of basic fish biology [9] and for the analysis of fish populations and reproduction dynamics setting management policies to maximize exploitation whilst protecting the spawning potential of the stocks [6]. At the same time, it must be considered that different methods to quantify egg production are used to estimate spawning stock biomass, towards the implementation of scientifically-based fisheries management [3, 37].

Further investigations are needed to understand the molecular mechanisms that govern transcription of 5S rRNA and accompanying genes during gametogenesis and sex differentiation in fish. In addition, according to the development stage of ovaries, it will be crucial to understand the relationship between the activation of Pol I and III and the levels of 5S and 18S rRNA. It is also necessary to explore the possibilities to enlarge the scope of teleost fish species (with different reproductive strategies, geographical and habitat distribution, developmental history…) where the 5S/18S rRNA index could be successfully used as in the present work. The present authors consider that the pattern of accumulation of these ribosomal molecules could define the quality of the spawned oocytes, as levels of 5S rRNA can also be measured in in them. In this way, 5S rRNA quantitative analysis could be useful also in determining the quality of reproductive females in aquaculture hatcheries. Finally, 5S/18S rRNA index could be also used to study the environmental xenoestrogenicity and identify intersex individuals, as 5S rRNA levels can identify the presence of oocytes (mainly previtellogenic) in gonads.

## Supporting Information

S1 Fig Total RNA electrophoresis and electropherograms of 9 blue whitings captured in May 2014. Samples 1 to 7 belonged to females during early oogenesis with previtellogenic oocytes. Only 2 male individuals were available (samples 8 and 9). 5S rRNA predominated in females, while peaks belonging to 18S and 28S rRNA were very small. L: RNA 6000 Nano Kit Ladder. Two micrographs representative of the ovaries and the testes (early gametogenic stages) in the fish studied are shown. Scale bars = 200 μm.
(TIF)

S2 Fig Total RNA electrophoresis and electropherograms of 9 bogues captured in May 2014. The band belonging to 5S rRNA was clearly observable in females (individuals 1 to 7), where nearly no 18S or 28S rRNA was observed. Only two males were available for the study (individual 8 and 9). All individuals were in an early gametogenic stage, as it could be observed in micrographs of a representative ovary and a testis. L: RNA 6000 Nano Kit Ladder. Scale bars: 200 μm.
(TIF)

S3 Fig 5S/18S rRNA index in testes and ovaries of anchovies from two samplings. 15 female and 16 male anchovies sampled during the spawning season in April and May 2012. Anchovies normally spawn on the weekly basis for a period of a few months. So upon capture females could present many mature close to hydration oocytes, as it was the case in the sampling carried out in April, or could be initiating a new maturation cycle, as it was the case of the ovaries in May. In this last case less mature oocytes were observed. The 5S/18S rRNA index distinguished ovaries according to the maturation stage; ovaries with less mature oocytes showing highest index values. Males of both samplings are combined to simplify the graph. Different capital letters indicate significant differences between sexes and different lower case letters indicate differences between the two analysed ovary stages (Mann-Whitney, p<0,005). Scale bars in both micrographs = 200 μm.
(TIF)

S1 Table Minimal data set used for the generation of Figs 2, 3 and S3 Fig. Data provided as required by the journal.
(PDF)

S2 Table Minimal data set related to qPCR analyses and used for the generation of Figs 3 and 4. Data provided as required by the journal.
(PDF)

S3 Table *gtf3a* sequences obtained from the different fish species studied. Table depicts size of amplified and sequenced fragments and e-value through BlastX analysis with the most similar sequences in Genbank. For comparative purposes the *Danio rerio gtf3ab* sequence (NP_001083013) appears in all the cases.
(PDF)
