# Supplementary figures and images for: Identification of Sex and Female’s Reproductive Stage in Commercial Fish Species through the Quantification of Ribosomal Transcripts in Gonads
Source: PLoS One. 2016 Feb 26;11(2):e0149711. doi: 10.1371/journal.pone.0149711 (PMC4771027; doi:10.1371/journal.pone.0149711)

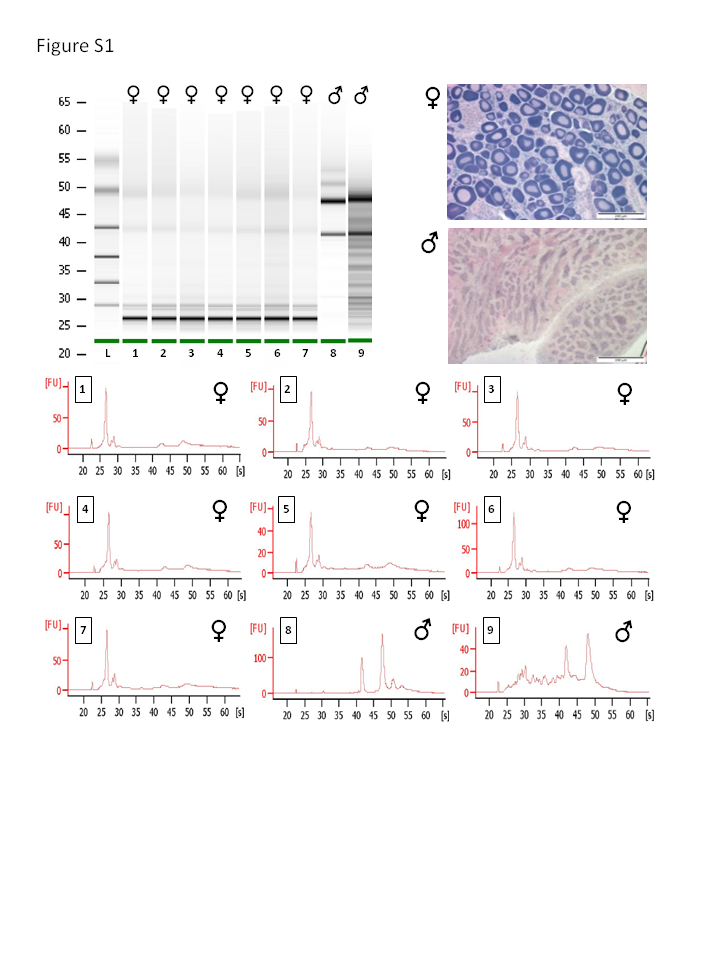

Supplement: S1 Fig — Samples 1 to 7 belonged to females during early oogenesis with previtellogenic oocytes. Only 2 male individuals were available (samples 8 and 9). 5S rRNA predominated in females, while peaks belonging to 18S and 28S rRNA were very small. L: RNA 6000 Nano Kit Ladder. Two micrographs representative of the ovaries and the testes (early gametogenic stages) in the fish studied are shown. Scale bars = 200 μm. (TIF) [file pone.0149711.s001.tif]

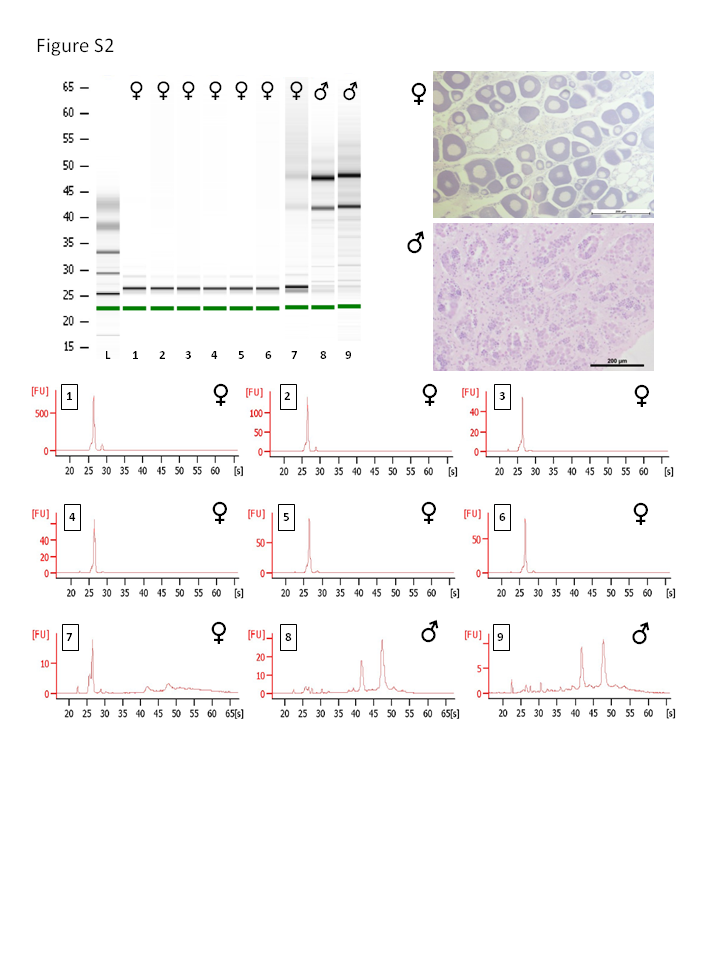

Supplement: S2 Fig — The band belonging to 5S rRNA was clearly observable in females (individuals 1 to 7), where nearly no 18S or 28S rRNA was observed. Only two males were available for the study (individual 8 and 9). All individuals were in an early gametogenic stage, as it could be observed in micrographs of a representative ovary and a testis. L: RNA 6000 Nano Kit Ladder. Scale bars: 200 μm. (TIF) [file pone.0149711.s002.tif]

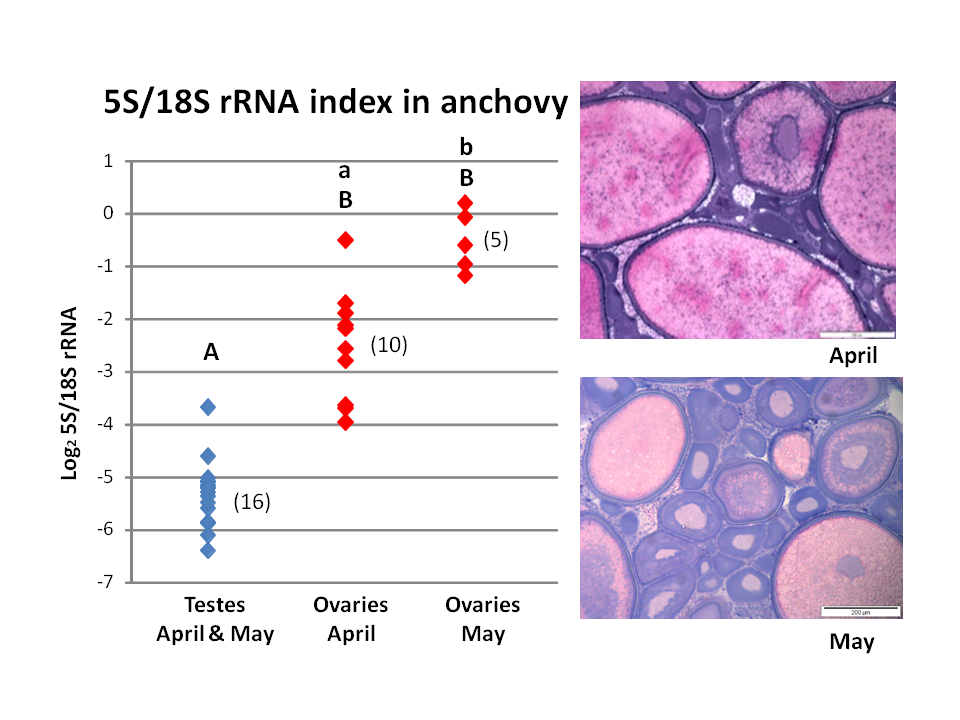

Supplement: S3 Fig — 15 female and 16 male anchovies sampled during the spawning season in April and May 2012. Anchovies normally spawn on the weekly basis for a period of a few months. So upon capture females could present many mature close to hydration oocytes, as it was the case in the sampling carried out in April, or could be initiating a new maturation cycle, as it was the case of the ovaries in May. In this last case less mature oocytes were observed. The 5S/18S rRNA index distinguished ovaries according to the maturation stage; ovaries with less mature oocytes showing highest index values. Males of both samplings are combined to simplify the graph. Different capital letters indicate significant differences between sexes and different lower case letters indicate differences between the two analysed ovary stages (Mann-Whitney, p<0,005). Scale bars in both micrographs = 200 μm. (TIF) [file pone.0149711.s003.tif]
