# Supplementary material for: Identification of Sex and Female’s Reproductive Stage in Commercial Fish Species through the Quantification of Ribosomal Transcripts in Gonads
Source: PLoS One. 2016 Feb 26;11(2):e0149711. doi: 10.1371/journal.pone.0149711 (PMC4771027; doi:10.1371/journal.pone.0149711)
Supplement: S1 Table — Data provided as required by the journal. (PDF) [file pone.0149711.s004.pdf]

Data used in:	Figure 2. Figure 3. Suplementary Figure 3

Species	5S rRNA T.C.A	18S rRNA T.C.A	Sex
Bogue	1379,4	3,4	F
	183,8	0,1	F
	1	13,1	M
	113,1	0,1	F
	168,7	0,1	F
	130,5	0,1	F
	250,3	0,1	F
	94,3	0,1	F
	85,5	1,3	F
Chub mackerel	6089,4	28,6	F
	6392,8	66,1	F
	37,6	240,1	M
	46	195,5	M
	3962,6	71,7	F
	3655,8	31	F
	7,6	78,3	M
	2,1	26,3	M
	126,2	0,1	F
	8,9	24,5	M
	4,7	9,2	M
	172,8	0,1	F
	170,5	1,9	F
	100,7	0,1	F
Hake	6796,7	304,2	F
	69	936,2	M
	5,4	34,8	M
	989,6	159,5	F
	6812,5	200,7	F
	6740,4	136,3	F
	4381,1	250,7	F
	13	50,8	M
	4639,3	306,8	F
	81	425,1	M
	5126,3	141,2	F
Blue withing	218,9	15,2	F
	251,9	18,2	F
	228,3	13,1	F
	231,5	8,8	F
	241,8	10,7	F
	229,1	13,3	F
	140,2	10,3	F
	279	12,4	F
	4,8	1539	M
	257,6	9,2	F
	283,9	13,8	F

M: testis

F: ovary

	4	3,3	M
	339,8	24,6	F
	293,2	15,8	F
	303,4	14,6	F
Megrim	226,17	40,7	F
	15,2	163,3	F
	27,4	111,2	F
	37,8	112,8	F
	225,2	75,9	F
	46	93,4	F
	403,2	95,5	F
	143	83,7	F
	62,2	222,2	F
	270,9	117,6	F
	0,2	14,4	M
	0,1	5,1	M
	0,1	16,5	M
	0,1	12,3	M
	487,8	22,2	F
	0,2	12,5	M
	431,4	20,1	F
	183,3	13,9	F
	136,7	18,8	F
	0,1	26,6	M
	10,2	37,7	F
	14,5	35,1	F
	21,2	25,3	F
	9,2	44,7	F
	10,8	24,4	F
	12,3	69	F
	12,6	32,4	F
	35,6	18	F
	9,5	37,6	F
	9,1	35,9	F
	25,7	8,1	F
	18,1	43	F
Horse mackerel	46,6	14,4	F
	86,1	5,8	F
	2,6	125,9	M
	4,1	141	M
	64	115,5	M
	3,3	90,7	M
	1,3	45,3	M
	3,6	82,9	M
	8,4	145,3	M
	2,2	94,8	M
	2,4	55,3	M
	5,3	67,8	M
	2,3	72,8	M
	135,1	44	F

	31,9	3,9	F
	4,8	30	M
	37,9	11,7	F
	4,2	22,2	M
	0,8	30,9	M
	44,8	3,3	F
	29,6	7,2	F
	1,7	38,2	M
	0,1	28,3	M
	3,9	31,1	M
Zebrafish	44,8	11,1	F
	70,5	9,2	F
	75,3	7,8	F
	15,4	7,7	F
	18,3	13,4	F
	8,4	8,7	M
	0,2	2,1	M
	4	7,8	M
	7,4	17	M
	9,2	23,1	M
Atlantic mackerel	6	83,6	M
	5,3	32,5	M
	16,1	138	M
	15,4	55,9	M
	64,9	32,9	F
	48,7	22,7	F
	31,4	18,4	F
	38,3	18,6	F
	21	110,5	M
	34,5	28,3	F
	102,9	91,3	F
	36,6	28	F
	9,5	49,8	M
Pilchard	143,2	80,7	F
	105,6	181,8	F
	67	84,7	F
	22,1	27,9	M
	20	144,3	M
	102,6	203,6	F
	0,8	148,8	M
	34,1	143,7	F
	18,6	145,2	M
	20,2	257,3	M
	93,6	164,9	F
	169,3	183	F
	2,8	43,3	F
	2,2	81,5	M
	7,4	91,5	F
	11,2	144,8	F
	17,6	79,5	F

Anchovy	1,3	76,2	M
	1,4	96,4	M
	1,1	92,2	M
	4,3	29,6	F
	10,3	44,6	F
	2,1	100,3	M
	3,6	120,6	M
	4,4	150	M
	25,6	150,6	F
	10,6	435,3	M
	20,5	28,8	F
	2,2	127,5	M
	13,8	44,7	F
	11,2	41,1	F
	39,4	34,1	F
	30,3	31,5	F
	1,8	80,2	M
	3,8	123	M
	2,8	108,6	M
	19,9	30,1	F
	3,9	138,6	M
	3,9	94,7	M
	13,2	77,5	M
	26,5	51,2	F
	3,6	131,4	M
	31	69,7	F
