# Supplementary material for: Identification of Sex and Female’s Reproductive Stage in Commercial Fish Species through the Quantification of Ribosomal Transcripts in Gonads
Source: PLoS One. 2016 Feb 26;11(2):e0149711. doi: 10.1371/journal.pone.0149711 (PMC4771027; doi:10.1371/journal.pone.0149711)
Supplement: S2 Table — Data provided as required by the journal. (PDF) [file pone.0149711.s005.pdf]

Data used in:	Figure 4.			Figure 3			

CT value		Sex/Ovarian developmental stage	CT value		CT value		Ovarian developmental stage
Sample ID	**gtf3a**		Sample ID	**5S rRNA**	Sample ID	**18S rRNA**	
GA1.1	23,942	CA	GA1.1	24,4323	GA1.1	24,4323	CA
GA1.1	23,858	CA	GA1.1	23,7287	GA1.1	23,7287	CA
GA1.1	23,881	CA	GA1.1	23,8812	GA1.1	23,8812	CA
GA1.2	26,975	AV	GA1.2	27,2492	GA1.2	27,2492	AV
GA1.2	27,095	AV	GA1.2	26,9709	GA1.2	26,9709	AV
GA1.2	27,031	AV	GA1.2	27,3482	GA1.2	27,3482	AV
GA1.3	27,179	AV	GA1.3	26,3295	GA1.3	26,3295	AV
GA1.3	27,093	AV	GA1.3	26,4795	GA1.3	26,4795	AV
GA1.3	27,088	AV	GA1.3	26,267	GA1.3	26,267	AV
GA1.4	26,052	AV	GA1.4	25,9339	GA1.4	25,9339	AV
GA1.4	25,853	AV	GA1.4	25,987	GA1.4	25,987	AV
GA1.4	25,965	AV	GA1.4	25,681	GA1.4	25,681	AV
GA1.5	22,704	CA	GA1.5	25	GA1.5	25	CA
GA1.5	22,942	CA	GA1.5	24,2357	GA1.5	24,2357	CA
GA1.5	22,882	CA	GA1.5	24,2243	GA1.5	24,2243	CA
GA1.6	26,217	AV	GA1.6	24,2143	GA1.6	24,2143	AV
GA1.6	26,461	AV	GA1.6	25,1292	GA1.6	25,1292	AV
GA1.6	26,334	AV	GA1.6	25,8508	GA1.6	25,8508	AV
GA1.7	23,937	CA	GA1.7	23,7096	GA1.7	23,7096	CA
GA1.7	23,968	CA	GA1.7	23,8189	GA1.7	23,8189	CA
GA1.7	23,940	CA	GA1.7	23,691	GA1.7	23,691	CA
GA1.8	27,898	AV	GA1.8	24,8731	GA1.8	24,8731	AV
GA1.8	27,972	AV	GA1.8	25,0821	GA1.8	25,0821	AV
GA1.8	27,818	AV	GA1.8	25,2639	GA1.8	25,2639	AV
GA1.9	24,207	CA	GA1.9	25,2212	GA1.9	25,2212	CA
GA1.9	24,339	CA	GA1.9	25,096	GA1.9	25,096	CA
GA1.9	24,387	CA	GA1.9	25,148	GA1.9	25,148	CA
GA1.10	25,965	AV	GA1.10	25,281	GA1.10	25,281	AV
GA1.10	25,989	AV	GA1.10	25,19	GA1.10	25,19	AV
GA1.10	25,783	AV	GA1.10	25,4884	GA1.10	25,4884	AV
GA1.11	24,274	CA	GA1.11	23,8574	GA1.11	23,8574	CA
GA1.11	23,877	CA	GA1.11	23,7112	GA1.11	23,7112	CA
GA1.11	23,880	CA	GA1.11	23,6446	GA1.11	23,6446	CA
GA1.15	26,035	AV	GA1.15	25,3821	GA1.15	25,3821	AV
GA1.15	26,267	AV	GA1.15	25,4842	GA1.15	25,4842	AV
GA1.15	26,080	AV	GA1.15	25,4976	GA1.15	25,4976	AV
GA2.1	40,000	M	GA2.5	22,9043	GA2.5	22,6119	PV
GA2.1	40,000	M	GA2.5	22,6119	GA2.5	22,4548	PV
GA2.1	40,000	M	GA2.5	22,4548	GA2.5	23,6164	PV
G2.2	37,453	M	GA2.11	23,7107	GA2.11	23,4811	PV
G2.2	38,355	M	GA2.11	23,4811	GA2.11	23,6266	PV
G2.2	37,456	M	GA2.11	23,6266	GA2.11	23,2903	PV
GA2.3	35,611	M	GA2.12	23,2903	GA2.12	23,8184	PV

GA2.3	36,672	M
GA2.3	36,683	M
GA2.4	36,032	M
GA2.4	37,480	M
GA2.4	36,057	M
GA2.5	22,302	PV
GA2.5	22,408	PV
GA2.5	22,407	PV
GA2.7	36,218	M
GA2.7	36,982	M
GA2.7	35,323	M
GA2.11	26,854	PV
GA2.11	26,744	PV
GA2.11	26,725	PV
GA2.12	24,115	PV
GA2.12	24,189	PV
GA2.12	24,055	PV
GA2.13	23,886	PV
GA2.13	24,021	PV
GA2.13	24,090	PV
GA2.14	35,465	M
GA2.14	35,347	M
GA2.14	35,087	M
GA3.8	23,707	CA
GA3.8	23,638	CA
GA3.8	23,523	CA
GA3.11	24,205	CA
GA3.11	24,380	CA
GA3.11	24,299	CA

GA2.12	23,8184	GA2.12	23,5948	PV
GA2.12		GA2.12	23,3668	PV
GA2.13	23,5948	GA2.13	23,5163	PV
GA2.13	23,3668	GA2.13	24,1797	PV
GA2.13	23,5163	GA2.13	23,8718	PV
GA3.8	24,4662	GA3.8	27,0896	CA
GA3.8	24,6346	GA3.8	27,1206	CA
GA3.8	25,229	GA3.8	27,0334	CA
GA3.11	26,4037	GA3.11	28,4954	CA
GA3.11	26,1496	GA3.11	27	CA
GA3.11	25,8247	GA3.11	28,5201	CA

M: testis	F: ovary
	PV:previtellogenic stage<br>CA: cortical alveoli stage<br>AV: advanced vitellogenesis
