# Supplementary material for: Identification of Sex and Female’s Reproductive Stage in Commercial Fish Species through the Quantification of Ribosomal Transcripts in Gonads
Source: PLoS One. 2016 Feb 26;11(2):e0149711. doi: 10.1371/journal.pone.0149711 (PMC4771027; doi:10.1371/journal.pone.0149711)
Supplement: S3 Table — Table depicts size of amplified and sequenced fragments and e-value through BlastX analysis with the most similar sequences in Genbank. For comparative purposes the Danio rerio gtf3ab sequence (NP_001083013) appears in all the cases. (PDF) [file pone.0149711.s006.pdf]

S3 Table. *gtf3a* sequences obtained from the different fish species studied. Table depicts size of amplified and sequenced fragments, and e-value through BlastX analysis with the most similar sequences in Genbank. For comparative purposes the *Danio rerio gtf3ab* sequence (NP_001083013) appears in all the cases.

**Species**	**Genbank accession number**	**Size (bp)**	**E-value**	**BlastX**<br>**Most similar sequence and Genbank accession number**
M. merluccius	JQ928630	523	3e-76<br>7e-54	*Oreochromis niloticus* PREDICTED TFIIIA,XP_003443591<br>*Danio rerio,* TFIIIA, b NP_001083013
S. scombrus	JQ928631	699	3e-133<br>5e-93	*Oreochromis niloticus* PREDICTED TFIIIA,XP_003443591<br>*Danio rerio,* TFIIIA, b NP_001083013
S. colias	JQ928632	707	2e-124<br>3e-96	*Oreochromis niloticus* PREDICTED TFIIIA,XP_003443591<br>*Danio rerio,* TFIIIA, b NP_001083013
L. whiffiagonis	JQ928633	691	2e-134<br>1e-93	*Oreochromis niloticus* PREDICTED TFIIIA,XP_003443591<br>*Danio rerio,* TFIIIA, b NP_001083013
S. pilchardus	JQ928634	636	6e-99<br>2e-91	*Danio rerio,* TFIIIA, b NP_001083013<br>*Oreochromis niloticus* PREDICTED TFIIIA,XP_003443591
M. poutassou	KC191719	594	2e-70<br>3e-46	*Takifugu rubripes*, PREDICTED TFIIIA like, XP 003968009<br>*Danio rerio,* TFIIIA, b NP_001083013
T. trachurus	KC191721	594	1e-112<br>2e-68	*Maylandia zebra,* PREDICTED TFIIIA like, XP 004546742<br>*Danio rerio,* TFIIIA, b NP_001083013
